# The Role of NK Cells in EBV Infection and Related Diseases: Current Understanding and Hints for Novel Therapies

**DOI:** 10.3390/cancers15061914

**Published:** 2023-03-22

**Authors:** Maria G. Desimio, Daniela A. Covino, Beatrice Rivalta, Caterina Cancrini, Margherita Doria

**Affiliations:** 1 Primary Immunodeficiency Research Unit, Bambino Gesù Children’s Hospital, IRCCS, 00165 Rome, Italy; 2 Department of Systems Medicine, University of Rome Tor Vergata, 00133 Rome, Italy

**Keywords:** Epstein–Barr virus, EBV, natural killer cell, NK cell, infectious mononucleosis, IM, EBV^+^ lymphoproliferative disease, EBV^+^ malignancy, inborn error of immunity, IEI, immunodeficiency, immunotherapy

## Abstract

**Simple Summary:**

Primary infection with the Epstein–Barr virus (EBV), one of the most common human viruses worldwide, mainly occurs in early childhood without causing symptoms but, especially when delayed into adolescence, it may lead to infectious mononucleosis (IM). Once acquired, EBV persists in latently infected B cells for life and, most importantly, is associated with several hematologic and epithelial tumors that preferentially develop in the context of immunodeficiency. Thus, understanding the interplay between EBV and the immune system is of great medical interest to drive the development of effective treatments, especially since vaccines and specific antivirals are still not available for EBV. Here we provide an overview of the actual knowledge on the role of natural killer (NK) cells in the control of EBV, summarizing in vivo and in vitro studies and also discussing possible employment of NK cell-based immunotherapies that have developed rapidly in recent years.

**Abstract:**

The Epstein–Barr virus (EBV) is a ubiquitous herpesvirus most often transmitted during infancy and infecting the vast majority of human beings. Usually, EBV infection is nearly asymptomatic and results in life-long persistency of the virus in a latent state under the control of the host immune system. Yet EBV can cause an acute infectious mononucleosis (IM), particularly in adolescents, and is associated with several malignancies and severe diseases that pose a serious threat to individuals with specific inborn error of immunity (IEI). While there is a general consensus on the requirement for functional CD8 T cells to control EBV infection, the role of the natural killer (NK) cells of the innate arm of immunity is more enigmatic. Here we provide an overview of the interaction between EBV and NK cells in the immunocompetent host as well as in the context of primary and secondary immunodeficiencies. Moreover, we report in vitro data on the mechanisms that regulate the capacity of NK cells to recognize and kill EBV-infected cell targets and discuss the potential of recently optimized NK cell-based immunotherapies for the treatment of EBV-associated diseases.

## 1. Introduction

Natural killer (NK) cells are the major cellular components of innate immunity endowed with the intrinsic capacity to detect and lyse aberrant cells, such as virus-infected and cancer cells. As extensively described in recent reviews [1,2], the cytolytic activity of NK cells does not rely on antigen-specific recognition and is regulated by the balance of signals triggered by inhibitory and activating receptors upon binding to cognate ligands on the surface of target cells. Functionally, NK cells use the same killing mechanisms as CD8 T cells, which is release of cytotoxic granules containing apoptosis-inducing proteins, but may also initiate apoptosis of target cells by triggering their death receptors (TRAIL-R and CD95). In addition, upon target cell recognition or cytokine-mediated stimulation, NK cells secrete IFN-γ, TNF-α, GM-CSF, and other cytokines and chemokines that activate and attract T lymphocytes and various innate cells, hence promoting different immune responses. NK cells are primarily distinguished by the expression of the CD56 adhesion molecule and CD16 (low-affinity IgG Fc region receptor III, FcγRIII) that are present on the cell membrane with a variable density, with CD56^dim^CD16^+^ cells and CD56^bright^CD16^-/low^ cells representing >90% and about 5%, respectively, of the NK cell populations circulating in peripheral blood. NK cells are kept inactive by the interaction of their inhibitory receptors (inhibitory killer cell immunoglobulin-like receptors—iKIRs, NKG2A, LILRB-1) with their ligands belonging to the human leukocyte antigen class I (HLA-I) family of molecules present on healthy cells [3]. The inhibitory receptor/HLA-I interactions have a key role during NK cell ‘education’, a process through which NK cells become functionally competent and tolerant to the surrounding environment [3,4]. During malignant transformation and viral infection, partial or complete loss of HLA-I expression may occur as a strategy to evade CD8 T-cell recognition, thus favoring activation of NK cells in which inhibitory signals are missing (‘missing-self’ model) [5]. In addition, unhealthy cells upregulate or express de novo ligands for activating NK cell receptors (including NKp30, NKp44, and NKp46—referred to as natural cytotoxicity receptors or NCRs, NKG2D, DNAM-1, NKG2C), hence inducing NK cell recognition and killing (‘induced-self’ model) [1,3,4]. Of note, activating receptor/ligand pairs also mediate the capacity of NK cells to kill activated T cells, which could be relevant for the maintenance of lymphocyte homeostasis and in the context of autoimmunity or graft-versus-host disease [6]. Moreover, NK cells can kill antibody-coated cells via antibody-dependent cellular cytotoxicity (ADCC) triggered by their Fc receptor CD16.

The receptor repertoire changes during NK cell maturation [1,7]. Briefly, the less mature subset of CD56^bright^ cells, which are potent producers of inflammatory cytokines but poorly cytotoxic, display constitutively high expression of NKG2A, NKG2D, NKp30, and NKp46. During the transition from CD56^bright^ to highly cytotoxic CD56^dim^ mature cell populations, NK cells progressively acquire expression of CD16, KIRs, NKG2C, cytotoxic proteins, and, ultimately, the CD57 senescence marker, while they downregulate CD56 and lose NKG2A expression. During the course of chronic viral infections, expansion of dysfunctional NK cells that have lost CD56 expression have been described (referred to as CD56^−^ or CD56^neg^ NK cells) [8]. Additionally, under pathologic conditions, inhibitory checkpoints might be expressed and affect NK cell function against cognate ligand-expressing targets [3]. Finally, accumulating evidence indicates that some viruses, such as the human cytomegalovirus (HCMV), can induce the clonal expansion of long-lived NK cell subsets endowed with adaptive features, including epigenetic reprogramming, peculiar changes in the receptor repertoire, and superior virus-specific effector functions as compared with conventional NK cells; henceforth, they have been defined ‘memory-like’ NK cells [9,10].

The importance of NK cells in the control of the Epstein–Barr virus (EBV) is strongly supported by the fact that patients with selective NK cell deficiency exhibit high susceptibility to this pathogen [11,12]. EBV is an oncogenic γ-herpesvirus with a large double-stranded DNA genome (encoding more that 80 proteins and several non-coding RNAs) that infects over 95% of the word population, usually acquired during infancy as an asymptomatic infection and then carried for life in latently infected B cells under the control of the host immune system [13]. While the vast majority of infected individuals will never develop malignancies, various settings may perturb the EBV–host interaction allowing the virus to express its oncogenic potential. Indeed, it was estimated that EBV causes about 1.5% of all human tumors, being associated with a wide range of pathogenically distinct malignancies [14,15]. A key aspect of EBV biology is the dual strategy of replication: one modality is the release of viral particles by lytic replication, which is required for virus transmission to a different host, the other is viral DNA replication within latently infected cells, which guarantees virus propagation and persistence within the infected host. EBV infection starts with oral transmission via saliva exchange and proceeds through multiple steps, some of which are not fully understood, that have been described in relevant reviews [15,16,17]. In brief, EBV is transferred across the mucosal epithelium either via transcytosis or lytic replication in epithelial cells, and infects naïve B cells in the underlying lymphoid tissues; these infected B cells transiently express various lytic and latent EBV proteins (pre-latency), then the full viral latency transcription program is established (type III latency) in which eight proteins (EBNA-1, -2, -3A, 3B, -3C, -LP, LMP1, LMP2) and various non-coding RNAs (EBERs, BARTs, BHRF1) are expressed, causing cellular activation, proliferation, and resistance to apoptosis. Of great importance for experimental studies, lymphoblastoid cell lines (LCLs) with type III latency are readily established by infecting peripheral blood B cells with EBV in vitro. In vivo, presumably after activation from type III latency, surviving B cells enter the germinal center reaction and viral protein expression is restricted to EBNA1, LMP1, and LMP2 (type II latency); then, some of these cells differentiate into memory B cells that function as long-term EBV reservoirs, in which only EBNA1 (type I) or no viral gene (type 0) is expressed. As an alternative to this ‘germinal center model of EBV persistence’, a route to viral persistence in memory B cells without passing through latency III infection has been described (‘persistence without transformation’ model) [16]. Further differentiation of infected memory cells into plasma cells reactivates EBV lytic replication, hence allowing infection of epithelial cells from the basolateral membrane and, ultimately, shedding of new viral particles into the saliva for transmission. The switch from latent to lytic EBV infection is under the control of both cellular factors (e.g., signaling pathways, epigenetic regulation, stress conditions) and viral gene products [18]. Upon reactivation, the immediate early BZLF1 and BRLF1 proteins are expressed and transactivate various early genes required for EBV DNA replication; then, late lytic proteins are synthetized, including capsid antigen and glycoproteins, followed by viral genome encapsidation and release of progeny virions.

EBV entry into B and epithelial cells is mediated by complex interactions between viral glycoproteins and cell-specific receptors; in addition, EBV can enter in atypical cell targets, such as NK, T, and smooth muscle cells, through molecular mechanisms that have not yet been identified [19]. All types of EBV-infected cells can undergo malignant transformation and develop into different forms of cancer, each associated with specific viral gene expression programs [20]. Various latent and lytic proteins as well as non-coding RNAs exert functions that could initiate and/or support cancer development, including pro-proliferative and anti-apoptotic activities, induction of genetic and epigenetic alterations, and evasion or suppression of anti-tumor immune responses [13,15,16].

In the immunocompetent host, EBV infection is kept in check by antiviral immune responses, particularly by virus-specific CD8 T cells that are strongly activated and expanded during primary infection, then, once viral load has dropped, persist at relatively high frequency as a long-lasting pool of memory cells [21,22]. In addition, EBV can be controlled by innate cytotoxic lymphocytes, namely, NK, iNKT, and γδ T cells, which are very abundant at the primary sites of viral entry, where they can eliminate infected cells and release immunostimulatory cytokines and chemokines before development of virus-specific adaptive T-cell responses [23,24]. Here we focus on the role of NK cells, reviewing multiple lines of in vivo and in vitro evidence indicating that these cells significantly contribute to the anti-EBV immune defense during primary infection as well as in the development of EBV-related diseases.

## 2. EBV-Related Lymphoproliferative and Malignant Diseases

Although the majority of EBV infections have an asymptomatic clinical course, several EBV-related pathologies can occur in patients with immunodeficiency, either primary or secondary, as well as in apparently normal individuals. A pathological response to EBV infection occurring in adolescents and young adults is infectious mononucleosis (IM), a self-limiting disease mainly characterized by prolonged fever, hepatosplenomegaly, lymphadenopathy, and CD8 T-cell lymphocytosis; in addition, IM has been linked to the increased risk of developing Hodgkin’s lymphoma (HL) and multiple sclerosis (MS) [25]. Moreover, EBV is associated with various lymphoproliferative disorders (LPDs) affecting B, NK, and T cells as well as non-hematopoietic malignancies (listed in Table 1) and may trigger hemophagocytic lymphohistiocytosis (HLH) or autoimmune diseases in patients with predisposition, malignancy, or rheumatologic disorder [26,27,28]. These conditions can arise from impaired immune surveillance of EBV, EBV-mediated immune evasion, dysregulated cytotoxic and inflammatory responses to EBV-infected cells, latent EBV infection of unnatural cell targets, or from other viral and host mechanisms not yet identified [22].

## 3. Complex EBV Infection in the Context of Immunodeficiency

The incidence of EBV-associated LPDs is greatly increased in individuals who are in a state of immunodeficiency due to specific therapies, co-infections, or inborn error of immunity (IEI). Specifically, patients who receive immunosuppressive therapy in the settings of solid organ transplantation (SOT) or hematopoietic stem cell transplantation (HSCT) may develop a series of post-transplant lymphoproliferative disorders (PTLDs) that are mostly associated with EBV infection and range from benign hyperplasia to malignant lymphoma [29,30]. The degree of immunosuppression, especially the loss of CD8 T-cell cytotoxic function against EBV, is considered a major determinant of PTLDs. Furthermore, immunosuppressive drugs used to treat autoimmune diseases were linked to the development of EBV-positive lymphoproliferative lesions [31,32]. Moreover, in patients with chronic HIV infection, which results in CD4 T-cell loss and reduced anti-viral function of CD8 T and NK cells, uncontrolled proliferation of EBV-infected lymphocytes may occur; accordingly, most HIV-associated LPDs display a plasma cell phenotype and are linked to EBV infection [33]. Moreover, simultaneous infection with *Plasmodium falciparum* or Kaposi sarcoma-associated herpesvirus (KSHV) results in higher EBV-positivity of LPDs in coinfected patients, which has been attributed to the capacity of the coinfecting pathogens to reduce the anti-EBV function of T and NK cells and to directly stimulate the pathogenic potential of EBV [34]. Of great clinical importance, individuals affected with various IEI can develop severe or even fatal EBV-induced diseases, an area of extensive investigation that has produced considerable information on the aspects of immunity that crucially control EBV infection. As thoroughly discussed in previous reviews [28,35,36,37], mutations in genes essential for CD8 T-cell activation/proliferation (*CTPS1, RASGRP1, ITK, CD27, CD70, TNFRSF9, MAGT1, CARMIL2*), cytotoxicity (*SH2D1A, CD27, CD70, TNFRSF9, MAGT1, CARMIL2, CORO1A, PI3KCD*), or survival (*XIAP, CORO1A, PI3KCD*) are associated with chronic EBV viremia in either 100% or in a significant fraction of affected individuals and with one or more EBV-related diseases, whose nature and frequency vary in an IEI-specific manner. Overall, IEI causing defective expansion of EBV-specific CD8 T cells promote the occurrence of lymphomas, whereas IEIs in which impaired killing of EBV-infected cells leads to protracted T-cell activation and expansion result in an increased risk of developing HLH [38]. The abovementioned IEIs also impair the function of NK cells and are mostly associated with reduced frequency of NK and/or iNKT cells, indicating that cell-mediated innate immunity has an important role in the control of EBV infection [23,39]. In addition, other IEIs affecting innate and/or adaptive immune cells and predisposing to multiple viral and non-viral infections may result in severe EBV infection, including mutations in *GATA2, MCM4, FCGR3A, CARD11, ATM*, and *WAS* [40,41,42,43,44,45]. Apparently, selective immunodeficiencies affecting B-cell function (e.g., CD40, CD19, CD81 deficiencies) or antigen-dependent CD4 T-cell activation (i.e., HLA-II deficiency) do not predispose to severe EBV infection, suggesting that these immune functions are not essential for EBV control [38].

## 4. Role of NK Cells in Primary Immunodeficiencies Predisposing to EBV-Induced Diseases

Studies on primary immunodeficiencies susceptible to EBV-induced diseases have provided important information about mechanistic requirements for EBV immune control that are shared by all cytotoxic lymphocytes, as reported in previous notable reviews [38,39]. In particular, impaired anti-EBV cytotoxic responses of both CD8 T and NK cells result from the loss of activities required for stimulatory signals (as in mutated *ITK, CD27, CD70, TNFRSF9, MAGT1, SH2D1A*, and *CARMIL2*), for actin remodeling and lytic granule dynamics at the immunological synapse (occurring in mutated *WAS*, *DOCK8*, *RASGRP1*, *CORO1A, CARMIL2* and, with gain-of-function mutation, *PI3KCD*), and for preventing immune senescence (e.g., in mutated *PI3KCD*). Interestingly, molecular studies on IEI due to mutations in *MAGT1* (referred to as ‘X-linked MAGT1 deficiency with increased susceptibility to EBV-infection and N-linked glycosylation defect’, XMEN), *CD70*, and *CARMIL2* have demonstrated that these three distinct diseases share a common reduction of the activating NKG2D receptor and, hence, of the cytotoxic activity of CD8 T and NK cells [46,47,48]. Apparently, downregulation of NKG2D is due to different causes, with the receptor being either destabilized by the loss of MAGT1-dependent glycosylation (in part also affecting CD70) [46] or, presumably, poorly expressed when co-stimuli are missing (i.e., CD70 and CARMIL2-mediated CD28 co-stimulation); notably, rescue of NKG2D expression and NKG2D-mediated cytotoxicity against EBV^+^ cell targets could be obtained by exposing MAGT1-deficient NK and CD8 T cells to Mg^2+^ in vitro [46], although Mg^2+^ supplementation in XMEN patients was not effective in a small study recently reported [49]. The simultaneous impairment of T and NK cells occurring in several IEIs predisposing to severe EBV infection does not allow discerning the individual role of each cell type in the immune control of the virus. Moreover, there is an open discussion about the essential vs. redundant role of NK cells in host defense against EBV and, more generally, in human immunity [50,51]. On the one hand, a nonessential role for NK cells was put forward because T^−^NK^−^ patients with severe combined immunodeficiency (SCID) who recovered T but not innate lymphoid cells after HSCT were not susceptible to severe EBV infection over a long follow-up period [52]; a further argument is that patients with IEIs that affect NK cell development and/or function (i.e., mutated *GATA2*, *MCM4*, *RTEL1*, *GINS1*, *IRF8*, and *FCGR3A*) and predispose to severe/fatal herpesvirus infections [12] might have defects in other immunological compartments, at least in some cases [38,51]. On the other hand, some evidence points to a non-redundant role of NK cells in controlling viral infections in SCID patients after HSCT [53]; moreover, the inverse correlation between NK cell numbers and disease progression in GATA2-deficient patients underlies the importance of these cells in the immune defense against herpesviruses [54,55].

## 5. Modulation and Function of NK Cells during EBV Infection

### 5.1. NK Cells in the Context of Primary EBV Infection

Initial evidence that NK cells play a role in EBV immunity was provided by studies in adolescents undergoing primary EBV infection, though opposite findings have been reported. Williams and colleagues described the transient expansion of NK cells with enhanced cytotoxic activity at diagnosis of acute IM [56]; since NK cell numbers were higher in patients with milder symptoms and NK cell frequency was inversely correlated with EBV viremia in the overall study population, the authors concluded that the magnitude of NK cell responses controls the clinical outcome of EBV infection. In contrast, a large prospective study in undergraduate students showed a positive correlation between NK cell expansion and viral load during acute EBV infection, with viral load also being associated with disease severity, hence indicating that symptoms might worsen via the exacerbated activation of NK (as well as CD8 T) cells [57]. In a longitudinal study on pediatric IM patients (age range of 2–15 years), Azzi and colleagues did not find an association between overall NK cell counts/frequency but identified a subset of NK cells with an early differentiated phenotype (CD56^dim^NKG2A^+^KIR^−^CD57^−^) that proliferated during the acute symptomatic phase in a manner directly correlated with cellular EBV DNA copies, exhibited an in vitro degranulation activity against autologous LCLs, and persisted for six months at higher frequency if compared to healthy or EBV-negative children with IM-like diseases [58]. The authors also showed that, in the healthy population, the frequency of CD56^dim^NKG2A^+^KIR^−^CD57^−^ cells is high at birth but then is continuously reduced during the first decade of life, a phenomenon attributed to the progressive differentiation of these cells into more mature NK cell subsets (via reduced expression of NKG2A, acquisition of KIRs and, ultimately, of the CD57 maturation marker) driven by exposure to common infections and other immunologic challenges [59,60]; thus, age-dependent loss of EBV-reactive early differentiated NK cells was suggested to cause the increased risk of IM in infected adolescents/adults. These results confirmed and extended a previous study in a humanized mouse model in which EBV infection induced expansion of NKG2A^+^KIR^−^ NK cells with antiviral activity before initiation of CD8 T-cell responses; most importantly, upon depletion of NK cells, infected mice showed higher viral titers, more severe IM symptoms (e.g., lymphocytosis, high INF-γ levels), and higher incidence of EBV-induced malignancies, indicating that early differentiated NK cells crucially restrict EBV infection and prevent disease progression [61]. Of note, mice infection with a mutated EBV unable to switch to lytic replication (BZLF-1-deficient) did not elicit activation of protective NK cell responses, indicating that these are not triggered during latency; however, NK cell depletion still resulted in higher T-cell activation, suggesting that NK cells prevent massive T-cell expansion and the consequent IM symptoms not only by limiting viral antigen loads, but also through cytotoxic restriction of activated T cells. Therefore, NK cells may control EBV infection both by killing infected cells and through their immunoregulatory capacity to suppress deleterious T-cell hyper-activation.

At present, the role of differentiated NK cells in EBV infection is unclear. Another recurrent herpesvirus, HCMV, induces the expansion of a terminally differentiated CD56^dim^NKG2C^high^KIR^+^ NK cell subset with adaptive ‘memory-like’ features that variably persists in HCMV-seropositive individuals and might protect from viral reactivation [62]. Apparently, this memory-like NK cell subset is specifically triggered by HCMV and not by EBV or HSV-1, although it can be further expanded in individuals co-infected with different viruses such as HIV, hantavirus, Chikungunya virus, and hepatitis viruses. Conversely, acute infection with EBV does not increase expansion of NKG2C^high^ memory-like NK cells in HCMV^+^ individuals but rather results in a higher frequency of mature CD56^dim^NKG2A^+^KIR^−^CD57^+^ NK cells persisting for at least 2.5 years as compared with EBV-infected HCMV^−^ individuals [63]. Whether these expanded CD56^dim^NKG2A^+^KIR^−^CD57^+^ cells have memory-like functions has not been investigated as yet. In general, the functional consequences of long-term changes induced by EBV in the NK cell compartment clearly deserve further examination.

Furthermore, reduced frequency of circulating immature CD56^bright^ NK cells was found in the acute or both acute and latent phases of EBV infection [58,63], which may reflect their maturation into early differentiated NK cells as well as their recruitment into secondary lymphoid organs. Indeed, studies on tonsillar NK cells, which are mainly CD56^bright^ as opposed to circulating blood NK cells, demonstrated that a subset of CD56^bright^ NK cells with an immature phenotype (NKG2A^+^CD94^+^CD54^+^CD62L^−^) accumulated in the tonsils of EBV^+^ individuals and, once activated and tested by in vitro assays, displayed a superior capacity to release IFN-γ and restricted more efficiently both EBV infection and tumorigenic transformation of B cells if compared to other tonsil-derived or blood NK cell subsets [64,65,66]. Therefore, although their activation during primary EBV infection has not yet been demonstrated, tonsillar CD56^bright^NKG2A^+^IFN-γ^high^ NK cells might exert an important constraint at the viral entry site.

### 5.2. In Vitro Activation/Expansion of NK Cells Triggered by EBV-Infected Cells

In some studies, the phenotype and function of NK cells that respond to EBV infection was investigated in detail following in vitro cocultures. In Hatton et al., LCLs were generated from B cells of healthy donors and used to challenge IL-2-activated autologous NK cells in a 4 h coculture prior to assessing by multicolor flow cytometry the cell-surface markers of NK cells that reacted by degranulating and producing IFN-γ (CD107a^+^IFNγ^+^ double positive cells) [67]; in this manner, a population of CD56^dim^CD16^−^NKG2A^+^2B4^+^NKG2D^+^NKG2C^−^CD57^−^ cells was identified as the predominant NK cell subset that recognized and eliminated EBV^+^ B cells, in accord with the phenotype of early differentiated NK cells that accumulated during primary symptomatic EBV infection [58]. With the aim of expanding ex vivo NK cells with anti-EBV function to be used therapeutically, another group cultivated NK cells isolated from healthy donors in the presence of IL-2 and either with or without an irradiated LCL line, hence obtaining a median 850-fold and 14-fold NK cell expansion, respectively, after two weeks [68]. Analysis of LCL-expanded NK cells showed that they were highly functional, with an increased cytotoxic activity as compared with cells grown with IL-2 only, and displayed a higher frequency of cells expressing NKG2D, DNAM-1, NKp30, and NKp44 activating receptors as well as other functional markers (e.g., TRAIL, FasL) as compared with primary NK cells; moreover, in line with in vivo data, NKG2A^+^, CD16^−^, and CD57^−^ cells were enriched. In another elegant study, an EBV^+^ Burkitt’s lymphoma (BL) cell line with type I latency, Akata, or a derivative EBV^−^ Akata cell line, either untreated or induced to enter the lytic cycle by cross-linking the BCR with an IgG-specific antibody (EBVAkata IgG^+^), was used to stimulate HLA-I-matched PBMCs of healthy donors in the presence of IL-2; after 10 days of coculture, a higher increment of NK cell numbers was measured by flow cytometry in cultures with Akata cells (>60-fold expansion) as compared with Akata IgG^+^ or EBV^−^ Akata (both untreated or IgG^+^) cell co-cultures, indicating that NK cell proliferation was induced by EBV to a higher extent during latency than in the lytic cycle phase [69]. Also in these settings, the major population of expanded NK cells after stimulation with EBV had an early differentiated phenotype (CD56^dim^NKG2A^+^KIR^−^NKG2C^−^CD57^−^), expressed high levels of 2B4 NKG2D, DNAM-1, and lacked CD16. Of note, within the same work it was found that about one-half of the tested donors responded to EBV in vitro stimulation with the strong expansion not only of NK cells but also of Vγ9Vδ2 T cells with a similar phenotype; the different responses were evenly balanced and independent of sex, HLA type, or previous exposure to EBV or HCMV, suggesting that there is a selective advantage to maintaining two alternative innate immune strategies against EBV infection.

### 5.3. NK Cells in EBV-Related Lymphoproliferative Diseases and Cancer

Deleterious changes in the NK cell compartment were described in individuals who developed serious, often life-threating, EBV^+^ lymphoproliferations and tumors, pointing to an important role of the anti-EBV responses of NK cells during pathogenesis of these diseases. By investigating NK cells in a cohort of heart and lung transplant children (Tx), Wiesmayr et al. found that, notwithstanding similar overall frequency, NK cells of patients with EBV^+^ PTLD differed from those of asymptomatic patients with or without detectable EBV viremia and from healthy controls because of a higher frequency of CD56^dim^CD16^−^ cells (as reported in acute IM) and of the anergic CD56^−^CD16^+^ cell subset at the expense of CD56^dim^CD16^+^ cells, NKG2D and NKp46 downregulation, upregulation of the PD-1 inhibitory immune checkpoint, and functional impairment in response to stimulation with cytokines or LCL targets ex vivo [70]. A similar trend was observed in asymptomatic Tx children with very high EBV viral load, suggesting that elevated EBV challenge of NK cells resulted in their functional exhaustion, which contributed to the immunopathogenesis of PTLD in Tx children. Recently, in a large cohort of adult SOT recipients, both NK and T cells were analyzed, comparing patients diagnosed with EBV^+^ PTLD, EBV^−^ PTLD, and control PTLD-free transplanted patients [71]; this study showed that EBV^+^ PTLD patients presented a profound NK cell lymphopenia, a higher proportion of PD-1^+^ NK cells associated with EBV viral load, and EBV-specific CD8 T reduced in number and functionally exhausted. Apparently, alterations of NK cells in EBV^+^ PTLD seem to be related to the interaction of these cells with EBV in the context of reduced anti-EBV T-cell responses. Furthermore, a higher proportion of poorly cytotoxic CD56^−^CD16^+^ NK cells, characterized by low NKp46 and NKp30 and high inhibitory KIR3DL1 levels, was found in children with endemic BL (eBL) from malarious regions of Africa, particularly in those with high EBV loads, compared with healthy children with or without malaria exposure [72]. Importantly, alteration of NK cells was resolved in long-term eBL survivors, suggesting that the NK cell potential against tumors and viruses was maintained and, in theory, could be a promising target for immunotherapy. Additionally, comparison of EBV^+^ vs. EBV^−^ classical Hodgkin’s lymphoma (cHL) in newly diagnosed adult patients, all sharing EBV seropositivity and the same clinical parameters except for higher plasma EBV load in the first group, showed a reduced frequency of CD56^dim^CD16^+^ cells associated with impaired ADCC activity of EBV^+^cHL NK cells [73]. Other NK cell features, such as maturation pattern and cytotoxicity against cell targets, were similar in EBV^+^cHL and EBV^−^cHL, yet the frequency of anergic CD56^−^CD16^+^ cells was significantly higher than in healthy controls only in EBV^+^cHL patients. Moreover, reduced NK cell number and/or functionality were described in some but not all studies of EBV^+^ nasopharyngeal carcinoma (NPC) patients; hence, the role of NK cells in this disease is as yet unclear [74]. In sum, despite key information having been achieved in various EBV-induced diseases, a better understanding of the role of NK cells in each population of patients is needed in order to customize NK cell-based immunotherapeutic strategies.

## 6. NK/EBV-Infected Cell Interactions

Several experimental studies have investigated the molecular mechanisms that mediate NK cell recognition and killing of EBV-infected cells with the aim of identifying new therapeutic opportunities for EBV-induced diseases. Overall, there is a general agreement on the superior potential of NK cells against cells undergoing lytic EBV replication as compared with latently EBV-infected targets; yet, as discussed herein, the key molecular mechanisms are poorly understood and various experimental data are controversial, possibly because of differences in the employed experimental cell systems or methods.

### 6.1. NK Cell Recognition of HLA-I Molecules on EBV^+^ Cell Targets

EBV has evolved multiple strategies to elude the host immune system, including downregulation of the HLA-I/antigen presentation machinery (APM) that can allow infected cells to evade CD8 T-cell attack. During the viral lytic phase, when a large number of viral antigenic proteins are expressed, various EBV proteins act in concert to inhibit APM: the transporter associated with antigen processing (TAP) complex is inhibited by BNLF2a and BCRF1, while HLA-I is downregulated by the early lytic BGLF5 and BILF1 proteins acting at the mRNA and protein level, respectively, as well as by BDLF3, which induces HLA-I internalization and degradation in the late lytic phase [75,76,77,78,79]. Notably, reduced cell-surface HLA-I levels on productively EBV-infected cells elicits evasion of CD8 T cells but, at the same time, sensitizes for recognition and killing by NK cells, which are activated in the absence of inhibitory KIR/HLA-I interactions (‘missing self’ mechanism of NK-cell lysis). Indeed, by employing a BL cell line with type I latency (AKBM) or in vitro established LCLs (type III) as targets of NK cell-mediated killing in cocultures with NKL, an NK cell line, or primary IL-2-activated NK cells, it was reported that these latent targets were poorly lysed but, once EBV entered the lytic cycle upon stimulation, they were very efficiently killed [58,80]. In line with HLA-I levels taking part in the susceptibility to NK cell lysis, HLA-I molecules are highly expressed in latently infected B cells [58,81], reflecting the status of activated B-cell blasts as well as the AMP up-modulation activity of viral factors such as LMP1 [82], but then they are drastically downregulated when EBV switches to the lytic program [80]. However, several lines of evidence indicate that this model is simplistic and that EBV exerts a more complex regulation of NK cell function than controlling overall HLA-I levels. First of all, the major NK cell population reacting in IM patients during EBV lytic infection in mouse models as well as against LCLs in vitro consisted of early differentiated cells lacking expression of KIR receptors for HLA-I [58,61,63,67], suggesting that distinct, likely activating, NK cell pathways might be involved in recognition of EBV during latency as well as productive replication (as discussed below). Second, notwithstanding drastic HLA-I downregulation, infected cells in the late phase of the EBV lytic cycle become resistant to NK cell-mediated lysis through the antiapoptotic activity of the viral Bcl2 homolog BHRF1 [83]. Third, during malignant transformation of latently EBV-infected cells, HLA-I can be downregulated by activation of cellular oncogenic factors, as shown for c-Myc in the context of NPC [82]. Moreover, various viral non-coding microRNA molecules, which are expressed in any phase of the EBV life cycle, were shown to interfere both directly and indirectly with AMP, affecting particularly cell surface expression of HLA-B molecules that present immunodominant EBV epitopes [84]. Finally, adding to the overall complexity, *KIR* and *HLA* genes are highly polymorphic, with KIR/HLA pairs influencing the capacity to control infection with viruses, including EBV [85].

### 6.2. NKG2A/HLA-E Interaction in NK Cell-Mediated Killing of EBV^+^ Cell Targets

As mentioned earlier, among activated NK cells, those expressing NKG2A are armed with a superior cytotoxic activity against LCLs as compared with NKG2A^−^ cells, which is counterintuitive if considering that NKG2A is an inhibitory receptor that can impair NK cell-mediated killing. However, this apparent discrepancy might be explained by the complex regulation of NK cell inhibitory signals. While inhibitory KIRs recognize the polymorphic HLA-A,B,C molecules, NKG2A (forming NKG2A/CD94 dimers) binds to ‘non classical’ HLA-E molecules that display limited polymorphism and are usually occupied by HLA-I leader peptides. Both KIRs and NKG2A, upon binding of their cognate ligands, provide inhibitory signals and are involved in the education of NK cells, yet some important differences exist. NKG2A has a stronger impact in the licensing process than KIRs [86], and high surface expression of HLA-E is required for NKG2A to inhibit activated NK cells, whereas KIR inhibition follows a linear relation with HLA-A,B,C levels on target cells [87]. Accordingly, a recent study showed that, within IL-2-activated NK cells, NKG2A^+^ cells degranulated more vigorously than NKG2A^−^ cells against tumor cells with low HLA-E levels in a manner that was independent of KIR and HLA-I expression on NK and target cells, respectively, suggesting that better licensing by NKG2A overrides its inhibitory signaling, at least in these settings [88]. Therefore, this phenomenon might explain why LCLs in which HLA-E is relatively poorly expressed during latency [58,67] and downregulated following lytic EBV replication [80,89], and our unpublished data] are killed more efficiently by NKG2A^+^ than NKG2A^−^ IL-2-activated NK cells [58,67]. Accordingly, antibody-mediated block of NKG2A did not modify NK cell degranulation against autologous LCLs, indicating that this receptor was not signaling but rather identified the subset of reacting (highly licensed) NK cells [67]. Moreover, functional studies have demonstrated that peptides derived from both latent and lytic viral proteins can associate with HLA-E and prevent NKG2A binding, thus unleashing NKG2A^+^ NK cell activation [90,91].

### 6.3. NKG2D/NKG2DLs and DNAM-1/DNAM-1Ls Axes Modulated by EBV

Studies in IEI with mutated *MAGT1, CD70,* and *CARMIL2*, as discussed earlier, suggested that one important receptor mediating anti-EBV cytotoxic responses is NKG2D [46,47,48]. NKG2D is expressed by NK and CD8 T cells as well as by subsets of iNKT, γδ T, and CD4 T cells, and recognizes eight cell surface proteins (MICA, MICB, and ULBP1-6 proteins, referred to overall as NKG2D ligands or NKG2DLs) whose expression is highly restricted in normal cells but can be induced via stress pathways on transformed or infected cells; upon ligand engagement, NKG2D delivers a potent stimulatory signal to NK cells and a co-stimulus to other immune cells, thus activating immune effector functions against tumors and viruses [92]. DNAM-1 is another activating receptor expressed by NK and CD8 T cells, in addition to distinct cell types, that recognizes two cell adhesion molecules (DNAM-1 ligands or DNAM-1Ls), CD155 (also referred to as poliovirus receptor-PVR or nectin-like molecule 5), and CD112 (nectin-2 or herpes entry mediator B-HVEB), which are generally up-modulated in response to various cellular stresses; hence, they can trigger cytotoxic responses against cells undergoing tumor transformation or infection [93]. The importance of NKG2D- and DNAM-1-mediated immune responses is emphasized by the fact that several viruses have evolved complex evasion strategies to impede the expression of these receptors and/or their ligands, as described in previous reviews [94,95]. Various members of the NKG2DL and DNAM-1L families have been investigated using different cell systems (i.e., LCLs, EBV^+^ tumor cells, EBV^−^ tumor cell lines transduced with EBV genes, EBV-infected B cells) and methods (staining of cell surface ligands either with a specific antibody or recombinant receptor, analysis of ligand mRNA level); few of these studies also tested susceptibility to killing by NK cells with or without blocking NKG2D or DNAM-1 axes by adding a receptor- or ligand-specific antibody (Table 2). In a study published more than 20 years ago, it was demonstrated that EBV^+^ BL Daudi cells with type I latency, but not Raji cells (EBNA6-deficient EBV^+^ BL, type III), expressed high ULBP1 levels (not MICA, ULBP2-3) and were susceptible to NK cell-mediated lysis that could be inhibited by an anti-NKG2D or anti-ULBP1 blocking antibody [96]. More recently, Pappworth and colleagues analyzed the expression of most NKG2DLs (all but ULBP4-6) as well as DNAM-1Ls and other cell surface molecules in an EBV^+^ BL type I cell line, AKBM, with and without viral reactivation; they showed that, with the exception of CD48, which can bind the 2B4 stimulatory receptor, activating ligands were not expressed during latency, when cells were poorly recognized by NK cells from different sources (IL-2-activated primary NK cells or patient-derived NKG2A^+^ NK cell lines, NKL and DEL NK); conversely, lytic cycle-induced cells displayed ULBP1 and CD112 on their surface while CD48 levels were maintained and HLA-I (including HLA-E) was downregulated, and became highly susceptible to killing by NK cells in part mediated by NKG2D and DNAM-1, as shown with an anti-ULBP1 and anti-CD112 blocking antibody [80]. Although neither NKG2DLs nor DNAM-1Ls were investigated, two subsequent reports confirmed that AKBM cells were killed by NK cells (activated NK cells or NKL) only when latent EBV switched to the lytic cycle [58,83], with one study also showing that lysis specifically occurred in the early lytic phase and in a manner that was dependent on NKG2D and, to a lesser extent, DNAM-1 but independent of NKp46, as determined by a receptor-specific blocking antibody [83]. On the other hand, the same studies provided less consistent evidence on the activating receptor/ligand pairs involved in NK/LCL interaction. Azzi et al. analyzed some activating ligands in LCLs, thus showing that MICA and CD48 were expressed during latency and, upon spontaneous lytic cycle activation, MICA and CD48 were further up-modulated and expression of both DNAM1-Ls, CD112 and CD155, was induced; this was associated with inefficient degranulation of activated NK cells with a CD56^dim^NKG2A^+^KIR^−^ phenotype when challenged with LCLs, but lytic cycle-induced LCLs were not tested and an antibody blocking-specific receptor/ligand pair was not employed [58]. Conversely, Williams et al. found that none of several tested ligands (MICA, MICB, ULBP2, CD112, CD155) was expressed on LCLs with or without EBV reactivation, yet lytic cycle-induced LCLs were killed by NKL via DNAM-1 independent of NKG2D or NKp46 based on the impact of the receptor-blocking antibody, suggesting that binding of DNAM-1 to an as yet unidentified ligand crucially mediates cytotoxicity [83]. Another study, in which only CD48 expression was analyzed on non-stimulated LCLs whereas an anti-NKG2D or anti-2B4-blocking antibody was used in cytotoxicity assays, demonstrated that activated NK cells, mainly CD56^dim^NKG2A^+^ cells, mediated lysis of LCLs in part via NKG2D, while 2B4 was dispensable despite high CD48 levels on targets [67]. Additional scattered evidence supported the important role of the NKG2D/NKG2DLs axis in NK cell-mediated recognition of LCLs in the absence of EBV reactivation. Indeed, LCLs were shown to display high levels of MICB, as well as low but detectable MICA levels, in two studies, one also showing low ULBP4 expression and the contribution of NKG2D to the recognition/killing by EBV-specific CD8 T cells (while NK cell lysis was not investigated) [97,98]. Furthermore, unspecified NKG2DLs were detected by means of recombinant NKG2D-Fc staining on LCLs generated from healthy individuals or XMEN patients; importantly, NK and CD8 T cells with low NKG2D expression isolated from XMEN patients poorly recognized autologous LCLs unless effectors were previously exposed to Mg^2+^, which resulted in NKG2D up-modulation [46]. Induced NKG2DL expression has also been described in EBV-infected cells other than LCL or BL cells. For example, primary B cells infected with EBV in vitro displayed ULBP4 on their surface, and their killing by γδ T cells was inhibited by a ULBP4-blocking antibody [99]. To study EBV-driven lymphomagenesis, Zhang et al. generated mice with B cell-restricted expression of the viral LMBP1 oncogenic protein; upon T-cell depletion, these animals succumbed because of diffuse LMBP1^+^ lymphomas expressing high levels of Rae, the murine ULBP ortholog [100]. Apparently, NK cells were not critical for tumor surveillance in this model, despite their ability to kill LMP1^+^ lymphoma cells in vitro, a killing that was partially inhibited by an NKG2D-blocking antibody. Of note, the authors treated T cell-depleted LMP1^+^ mice with low doses of recombinant NKG2D-Fc, which can trigger complement- and cell-dependent cytotoxicity, significantly reducing tumor growth and prolonging survival; therefore, they suggested that optimized doses of NKG2D-Fc have great potential in the treatment of EBV-driven pathologies, such as EBV^+^ PTLD, which displayed high (unspecified) ligand expression when stained with NKG2D-Fc [100]. Other experimental in vitro studies investigated the function of individual EBV proteins or miRNA on NKG2DL expression, providing evidence for both up- and downregulation. By transducing DG75 EBV^−^ BL cells with a vector encoding for the early lytic BZLF1 protein, Williams et al. found that ULBP2 expression was induced at the transcriptional level (not MICA, MICB, or CD155) and cells became susceptible to NKL-mediated lysis [83]. On the contrary, expression in EBV^−^ gastric carcinoma (GC) cells or other epithelial cell carcinomas of the LMP2A protein that is important for maintaining EBV latency and inducing host cell transformation, had a dual effect on the expression of MICA and MICB: LMP2A, on the one hand, upregulated their transcripts and, on the other hand, decreased total protein amounts, which resulted in a net reduction of both ligands at the cell membrane, though the functional consequences were not investigated [101]. Another recent work, using primary B cells infected with EBV either wt or deficient for the expression of the EBNA1 oncoprotein as well as epithelial cell lines transduced with EBNA1-expressing or control vector, showed that EBNA1 downregulated ULBP1 and ULBP5 mRNA levels, likely by reducing activation of cellular stress responses and c-Myc, and protected newly infected B cells from NKG2D-dependent killing by NK cells [102]. Finally, two independent works showed that EBV encodes for two distinct microRNAs targeting NKG2DL mRNA molecules, both belonging to the BART family and expressed in all forms of latency as well as during virus replication. The first to be discovered by a bioinformatic and functional approach, miR-BART2-5p, bound selectively MICB mRNA blocking its translation, hence leading to downregulation of cell surface MICB levels; this mechanism was shown to occur also in LCLs (i.e., 721.221 type III cells) in which expression of miR-BART2-5p decoy transcripts (the so called ‘anti-microRNA sponge’) resulted in MICB up-modulation and enhanced NK cell-mediated killing [97]. More recently, expression in EBV^−^ NPC cells of miR-BART7, previously identified as a functional suppressor of TGF-β, was sufficient to reduce MICA mRNA and protein levels and to impair sensitivity to killing by the NK92 cell line [103].

Altogether, a growing body of evidence indicates that EBV up-modulates ligands for NKG2D and DNAM-1, possibly because of the activation of cellular stress pathways that positively regulate ligand expression, but, at the same time, it has developed countermeasures to impede cell surface ligand expression and avoid immune recognition, a dual strategy that evolved in other viruses as well [104]. The complexity of NKG2DL and DNAM1L regulation by EBV is only beginning to emerge, being mediated by different viral activities that act at a distinct ligand expression level (transcriptional or post-transcriptional) and exerting either an inhibitory (e.g., mir-BART2/7, LMP2, EBNA1) or activating (e.g., LMP1, BZLF1) function, apparently without a clear separation between negative and positive regulation based on latent vs. lytic programs of the EBV life cycle.

In sum, a noticeable amount of data point at a critical role of the NKG2D/NKG2DL axis and, although less investigated, of the DNAM-1/DNAM-1L axis in the host anti-EBV immune response. Further investigation is clearly needed, and particular critical aspects should be taken into consideration: (i) the EBV gene expression program; (ii) specific features of the infected cell that influence activating ligand expression (cell type, resting vs. activated state, degree of malignant transformation); (iii) the polymorphism of NKG2D and its ligands (particularly high for MIC proteins); multiple levels of ligand regulation including epigenetic and proteolytic shedding at the cell membrane.

### 6.4. NK Cell-Mediated ADCC against EBV^+^ Cells

Few studies have investigated the role of NK cell-mediated killing of EBV-infected cells via ADCC, the most recent showing that EBV^+^ serum triggered strong NK cell degranulation and cytokine production against EBV-infected cells in the lytic cycle displaying surface expression of late gp350/220 viral protein (i.e., stimulated AKBM cells) [105]. Very recently, a comprehensive antibody profiling study in college students followed from the acute IM stage to one year later demonstrated that antibodies against lytic (p47/54, gp350/220, VCA-p18) as well as latent (EBNA1) proteins induced minimal Fc-mediated NK cell activation (as compared with influenza antigens); even in a tested reference group of chronically EBV-infected individuals, only gp350/220-specific antibodies induced low level NK cell degranulation [106]. The authors suggested that EBV, by switching from lytic replication to latency, prevents the induction of lasting and highly functional antibodies and that the possibility of boosting antibody responses to EBV via therapeutic interventions such as vaccination should be explored.

## 7. Current Treatments for EBV-Induced Diseases and Therapeutic Applications of NK Cells

Surgery, chemotherapy, radiotherapy, and HSCT are the established strategies for the treatment of EBV-related malignancies, but they are all burdened by several complications or adverse events. Moreover, despite extensive investigations, antiviral drugs that could effectively inhibit EBV replication have not been identified as yet [107]. At present, no prophylactic or therapeutic EBV vaccine has been approved, though several new formulations are under investigation, including vaccines containing multiple antigens and synthetic mRNA vaccines [108]. Given these limitations, the employment of rapidly developing immunotherapies in combination with conventional treatment for EBV-driven tumors has grown markedly in recent years. In this context, monoclonal antibodies targeting B cells (e.g., Rituximab, Brentuximab) have proved their efficacy as first-line or rescue therapies in PTLD or diffuse large B-cell lymphoma (DLBCL) [109]. Moreover, antibody-mediated immune checkpoint inhibition (ICI), namely, blocking the PD-1/PD-L1 interaction, yielded very encouraging results for the treatment of EBV-related NK/T cell lymphoma, HLH, and NPC [110,111,112], a strategy that could be extended to other PD-1L^+^ LPDs driven by EBV [113]. In the last few years, several laboratories have demonstrated that adoptive T-cell therapy with ex vivo manipulated cytotoxic T lymphocytes, either autologous or donor-derived, targeting B-cell antigens (i.e., chimeric antigen receptor T cell, CAR-T) or EBV antigens (e.g., LCL-stimulated or T-cell receptor-engineered T cell, TCR-T) is an effective treatment for several EBV-associated malignancies, including HL, NK/T-cell lymphoma, PTLD, and NPC [114,115]. Additional studies are needed to test whether better clinical outcomes could be obtained by combining adoptive T-cell therapy with ICI or therapeutic vaccination. On the other hand, adoptive T-cell therapy presents some limitations, such as occurrence of escape mutations in EBV antigens, downregulation of HLA-I molecules in some EBV^+^ LPDs, or congenital defects in T-cell co-stimulatory pathways (e.g., patients with mutated *CD70*). In this context, several advantages of NK cells over T cells in adoptive cell therapies have been recognized, including low/absent risk for graft-versus-host disease (GVHD), cytokine release syndrome or neurotoxicity; enhanced alloreactivity under KIR mismatch with HLA ligands on cancer cells; additional CAR-independent mechanisms mediated by CD16 (ADCC) and other activating receptors elicit tumor killing by CAR-NK cells [116,117]. These considerations motivated the implementation of allogeneic NK cells in the treatment of different hematologic and solid tumors, including EBV^+^ malignancies, with initial studies indicating safety and efficacy and a number of clinical trials being underway [116,117]. Intensive work is currently ongoing to gain improved “off-the-shelf” allogeneic NK cells from different sources (peripheral, umbilical cord or placental blood, NK-92 cell line, pluripotent stem cells), avoiding the use of feeder cells to improve safety, inducing ectopic IL-15 expression to gain superior cytotoxicity and persistence, differentiating into ‘memory-like’ cells with higher effector functions, and engineering CAR targeting NK cell-activating ligands (e.g., CAR.NKG2D NK cells) [118]. Moreover, therapeutic potential can be enhanced by combining adoptive NK cell therapy with NK cell engagers (i.e., antibody-derived constructs engaging both tumor antigen and CD16, possibly also linked with IL-15), ICI, or antibody-targeting B cells or tumor antigens [117,117]. A phase I study with autologous expanded NK cells and Cetuximab (antibody-recognizing EGFR that is highly expressed on NPC cells) in patients with recurrent and/or metastatic NPC showed promising results, with three out of seven individuals protected from disease progression [119]. Over the next few years, results from several ongoing clinical trials will ultimately provide important information on the applicability of NK cell-based therapies in EBV-related diseases. Finally, we speculate that therapies based on NK cells may synergize with the ‘lytic induction’ or ‘oncolytic’ therapeutic approach, which involves pharmacologic EBV reactivation in latently infected cells to induce apoptosis and/or susceptibility to antivirals [120]. Notably, several EBV-reactivating drugs (e.g., histone deacetylase inhibitors, HDACis) have entered into clinical trials because of their capacity to up-modulate ligands for NK cell-activating receptors in cancer cells, especially NKG2DLs [121]; hence, high NKG2DL levels simultaneously induced by the reactivating drug and the lytic viral replication may potently sensitize EBV^+^ cells for killing by NK cells, particularly by adoptively transferred activated NK cells with enhanced NKG2D expression or CAR.NKG2D NK cells.

## 8. Conclusions and Perspectives

**Figure 1:**
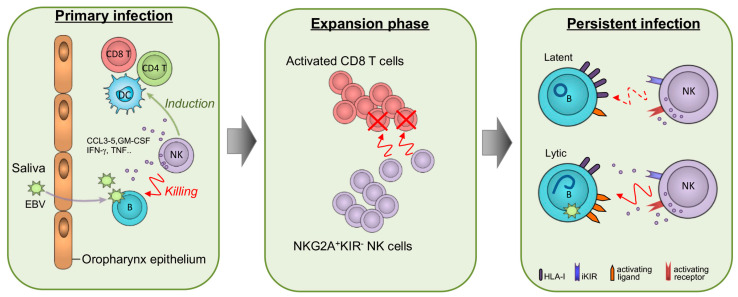
Schematic representation of NK cell functions in various phases of EBV infection. In secondary lymphoid tissues such as tonsils, NK cells exert a first-line defense against EBV that crosses the mucosal epithelium during primary infection (left panel); the antiviral functions of NK cells include direct killing of EBV-infected B cells, production of a wide range of cytokines (e.g., IFN-γ, TNF, and granulocyte/monocyte colony-stimulating factor, GM-CSF) that facilitate activation of T cells and innate immune cells such as dendritic cells (DCs), and secretion of chemokines that attract effector lymphocytes and myeloid cells at the sites of infection. During the acute phase of infection, a large expansion of activated CD8 T cells, many of which are EBV-specific, occurs coincidentally with the development of symptoms (expansion phase, central panel); in this phase, NK cells are also expanded, specifically an NKG2A^+^KIR^−^ NK cell population armed with the capacity to recognize and kill EBV-infected cells ex vivo and to eliminate highly activated CD8 T cells in vivo in a mouse model. In the infected host, EBV persists in latently infected B cells that are killed inefficiently by NK cells, possibly because they express high levels of HLA-I molecules recognized by inhibitory receptors (iKIR) and few/no NK cell-activating ligands; upon EBV reactivation and productive lytic replication, infected cells become highly susceptible to NK cell-mediated killing because of HLA-I downregulation and induction of ligands for activating receptors such as NKG2D.

In this review, we highlighted clinical and experimental evidence suggesting that NK cells are important in controlling EBV during all phases of the infection (Figure 1). In early primary infection, NK cells represent the first line of defense against EBV together with other innate cells (iNKT and γδ T cells) and help the priming of T-cell responses. Then, in the subsequent CD8 T-cell expansion phase, NK cells may restrain excessive immune activation by killing proliferating CD8 T cells, as indicated by humanized mouse studies. During latency, EBV-infected cells are likely to be poorly recognized by NK cells, yet they may become highly susceptible to NK cell-mediated killing as soon as the virus is reactivated as well as during EBV-induced malignant transformation, both settings in which HLA-I loss (associated with evasion of CD8 T-cell responses) and up-modulation of stress molecules recognized by activating NK cell receptors may occur. A deeper understanding of which NK cell subset is better suited for killing EBV^+^ cells in each viral expression program and of the underlying molecular pathways may uncover new opportunities for NK cell manipulation in innovative treatments for EBV-induced diseases.

## Figures and Tables

**Table 1:** EBV-associated lymphoproliferative disorders (LPDs) and malignancies.

*B-cell target*
Chronic active EBV (CAEBV) of B cells
Burkitt’s lymphoma (BL)
Hodgkin’s lymphoma (HL)
Diffuse large B-cell lymphoma (DLBCL)
Plasmablastic lymphoma
Lymphomatoid granulomatosis
Post-transplant lymphoproliferative disorders (PTLDs)
***NK or T-cell target***
CAEBV of NK or T cells
NK or T-cell lymphoma
***Epithelial cell target***
Nasopharyngeal carcinoma (NPC)
Gastric carcinoma (GC)
***Smooth muscle cell target***
Leiomyosarcoma

**Table 2:** Modulation of NK cell-activating ligands by EBV.

Cell System	EBV Effect on Activating Ligands for NK Cells	Impact on Immune Killing	Ref.
Daudi, Raji (EBV^+^ BL)	High ULBP1 expression (not MICA, ULBP2-3) in Daudi but not in Raji cells	Killing of Daudi but not Raji cells by activated primary NK cells was mediated by NKG2D/ULBP1 interaction (reduced by ULBP1 or NKG2D blocking Ab)	[96]
AKBM (EBV^+^ BL)	CD48 but not NKG2DL or DNAM-1L were expressed; ULBP1 and CD112 were induced upon lytic cycle induction (while CD48 was maintained)	Killing by NK cells (activated primary NK, NKL, and DEL NK) was low against latent AKBM (1–20%) but high against lytic AKBM (20–60%); NKG2D and DNAM-1 contributed to NKL lysis of lytic AKBM (reduced by ULBP1 or CD112 blocking Ab)	[80]
AKBM, LCL	Not tested in AKBM LCLs expressed MICA and CD48; upon lytic cycle activation, MICA and CD48 were up-modulated and CD155 and CD112 were induced	Cytotoxic degranulation of CD56^dim^NKG2A^+^KIR^−^ subset of activated NK cells was low against latent AKBM and LCLs but high against lytic AKBM	[58]
AKBM, LCL	Not tested in AKBM LCLs, either latent or lytic cycle-induced, did not express MICA, MICB, ULBP2, CD112, and CD155	NKL-mediated lysis was low/absent against latent (BZLF1^−^BcLF1^−^) or late lytic (BZLF1^+^BcLF1^+^) but high against early lytic (BZLF1^+^BcLF1^−^) AKBM or LCL cells; NKG2D and, to a smaller extent, DNAM-1 (not NKp46) contributed to killing of lytic AKBM while killing of lytic LCL was mediated by DNAM-1 (not NKG2D or NKp46 by blocking with receptor-specific Ab)	[83]
LCL	High CD48 expression	Lysis by autologous NK cells was low (5%) in part involving NKG2D but not 2B4 or NKG2A (as determined by Ab block); CD56^dim^NKG2A^+^ cells were more cytotoxic than CD56^dim^NKG2A^−^ cells	[67]
LCL (721.221)	High MICB expression (despite translational repression by miR-BART2-5p), low MICA levels	NK cell lysis was enhanced upon MICB up-modulation by transduction with anti-miR-BART2-5p ‘sponge’	[97]
LCL	High MICB expression, low MICA and ULBP4 levels because of LMP2A-mediated downregulation	NKG2D contributed to recognition by EBV-specific CD8 T cells (in part reduced via NKG2D Ab block); NK lysis not tested	[98]
LCL from XMEN patients	Unspecified NKG2DL expression (NKG2D-Fc staining)	Impaired killing by autologous NK or CD8 T cells unless NKG2D expression was restored	[46]
Murine LMP1^+^ B-cell lymphoma	Rae-1 (murine ULBP ortholog) was induced	NKG2D contributed to NK cell lysis (in part reduced via NKG2D Ab block) of LMP1^+^ lymphoma cells in vitro; treatment with NKG2D-Fc reduced tumor growth in transgenic LMP1^+^ mice	[100]
EBV^+^ PTLD	Unspecified NKG2DL expression (NKG2D-Fc staining)	Not tested	[100]
B cells infected with EBV	ULBP4 was induced by EBV infection	ULBP4 mediated killing by γδ T cells (halved by ULBP4 Ab block); NK lysis not tested	[99]
B cells infected with EBV	ULBP1 and ULBP5 mRNA levels were reduced by EBNA1	NK cells killed more efficiently and in an NKG2D-dependent manner (reduced via NKG2D Ab block) targets infected with EBNA1-deficient EBV as compared with wt virus	[102]
BZLF1^+^ DG75 (EBV^−^ BL)	BZLF1 expression in DG75 cells induced expression of ULBP2 at the transcriptional level (not MICA, MICB, or CD155)	NKL cells killed efficiently BZLF1^+^ULBP2^+^ DG75 but not control DG75 cells	[83]
miR-BART7^+^ EBV^−^ NPC	MICA downregulated via translational repression by miR-BART7	Lysis by the NK92 NK cell line against miR-BART7^+^ cells was reduced as compared to untreated cells	[103]
LMP2^+^ EBV^−^ GC	LMP2 expression down-modulated MICA and MICB despite increased mRNA levels	NK lysis not tested	[101]

Ab, antibody; BL, Burkitt’s lymphoma; GC, gastric carcinoma; LCL, lymphoblastoid cell line; NPC, nasopharyngeal carcinoma; PTLD, post-transplant lymphoproliferative disorder; wt, wild type; XMEN, X-linked MAGT1 deficiency with increased susceptibility to EBV infection and N-linked glycosylation defect.

## Data Availability

Not applicable.
